# Fluorescence-based super-resolution-microscopy strategies for chromatin studies

**DOI:** 10.1007/s00412-023-00792-9

**Published:** 2023-03-31

**Authors:** Thomas C. Q. Burgers, Rifka Vlijm

**Affiliations:** Molecular Biophysics, Zernike Institute for Advanced Materials, Rijksuniversiteit Groningen, Groningen, the Netherlands

**Keywords:** Chromatin organisation, Super-resolution microscopy, STimulated Emission Depletion microscopy (STED), STochastic Optical Reconstruction Microscopy (STORM), Chromatin labelling

## Abstract

Super-resolution microscopy (SRM) is a prime tool to study chromatin organisation at near biomolecular resolution in the native cellular environment. With fluorescent labels DNA, chromatin-associated proteins and specific epigenetic states can be identified with high molecular specificity. The aim of this review is to introduce the field of diffraction-unlimited SRM to enable an informed selection of the most suitable SRM method for a specific chromatin-related research question. We will explain both diffraction-unlimited approaches (coordinate-targeted and stochastic-localisation-based) and list their characteristic spatio-temporal resolutions, live-cell compatibility, image-processing, and ability for multi-colour imaging. As the increase in resolution, compared to, e.g. confocal microscopy, leads to a central role of the sample quality, important considerations for sample preparation and concrete examples of labelling strategies applicable to chromatin research are discussed. To illustrate how SRM-based methods can significantly improve our understanding of chromatin functioning, and to serve as an inspiring starting point for future work, we conclude with examples of recent applications of SRM in chromatin research.

## Introduction

The 3D organisation of chromatin is vital for gene regulation and cell fate. Chromosomes spatially organise into compartments during interphase, often referred to as chromosome territories (Cremer et al. 2006). Evaluating the level of organisation at the megabase length scale, chromatin regions can be considered to reside in an inactive, tightly packed state (heterochromatin) or in an active, less compacted state (euchromatin). Using 3C methods (including Hi-C), chromatin interactions can be mapped (Dekker et al. 2002; Lieberman-Aiden et al. 2009), and a 3D model of the genome can be reconstructed (Oluwadare et al. 2019) up to a resolution of 1 kb (Rao et al. 2014) (typical ~ 25–40 kb (Zhang et al. 2018)). These high-throughput methods have revealed among others the existence of topological associating domains (TADs) (Dixon et al. 2012) and chromatin loops (Rao et al. 2014). Although a powerful approach to analyse the interactions among specific DNA regions, this 3D reconstruction does not directly provide the physical compaction or location of chromatin regions, nor the location of proteins in these regions. Furthermore, the required sample processing inhibits live-cell measurements. Where the physical size of periodic chromatin structures can be obtained with high precision through small angle X-ray scattering (SAXS) (Joti et al. 2012; Langmore and Paulson 1983; Nishino et al. 2012; Sperling and Tardieu 1976), this method detects periodic structures of bulk chromosome samples in solution without the spatial information of individual structures in the nucleus. Both the interaction and the SAXS studies therefore can be complemented with imaging approaches to directly visualise the organisation of these structures within the nucleus. The best spatial resolution can be obtained using electron microscopy (EM), which revealed the nanoscale organisation of chromatin (Ou et al. 2017) and the chromatin hierarchy from the 10-nm beads on a string up to the 300–700-nm chromatids during mitosis (Eltsov et al. 2008; Finch and Klug 1976; Maeshima et al. 2010; McDowall et al. 1986; Olins and Olins 1974). The drawbacks of this contrast-based method, however, are the lack of multi-colour labelling, a relatively low throughput, and the necessity for cell fixation which prevents the detection of dynamic rearrangements.

Fluorescence-based microscopy uniquely does allow to obtain spatial information in (living) cells with high molecular specificity. Both chromatin and chromatin-associated proteins can be localised simultaneously using multi-colour labelling. However, the resolution of conventional light microscopy is diffraction limited (> 200 nm), only allowing to spatially resolve the overarching chromatin organisation. With the rise of super-resolution microscopy (SRM) methods, it became possible to generate images with a resolution that surpasses the diffraction limit, awarded with the 2014 Nobel Prize in Chemistry. Owing to recent developments in the microscope engineering, labelling, and analysis, SRM is now a viable method to unravel chromatin organisation, as it (i) can resolve chromatin structures at the level of a single or a few nucleosomes; (ii) has a high molecular specificity; (iii) allows for multi-colour imaging; and (iv) depending on the method can be live-cell compatible. Therefore, SRM seamlessly complements the other applied methods in chromatin organisation studies.

The aim of this review is to introduce the field of SRM to enable an informed selection of the most suitable SRM method for a specific chromatin-related research question. To date, there is a large variety of SRM techniques, each with its (dis)advantages. Of all the techniques that obtain an improved resolution compared to the diffraction limit, we focus on the two diffraction-unlimited SRM approaches: (i) coordinate-targeted SRM and (ii) stochastic-localisation-based SRM. We refer to other reviews for an in-depth discussion of the techniques that have an improved, yet still limited, resolution compared to confocal microscopy (e.g. structured-illumination microscopy, AiryScan, and lattice light-sheet microscopy) (Bond et al. 2022; Flors and Earnshaw 2011; Schermelleh et al. 2019). These techniques might be worth considering if their resolutions are sufficient and their specific advantages more important for the research question.

Of both the diffraction-unlimited SRM approaches, we will discuss the fundamental principles and highlight the advantages and disadvantages, including the spatial and temporal resolution, the live cell compatibility, the availability of multi-colour imaging, and the complexity of data collection and data processing. Due to the importance for the applicability, and the final obtained resolution, additionally we will discuss labelling and sample preparation strategies for chromatin and chromatin-binding proteins. We conclude with recent examples of SRM based chromatin research to illustrate the type of questions each SRM method can address and to serve as inspiration on how the direct visualisation of the spatial chromatin organisation can contribute to a holistic understanding of chromatin functioning.

## Coordinate-targeted super-resolution microscopy

### Technique

Fluorescence microscopy is very specific, as only the molecules of interest labelled with fluorophores (e.g. dyes or fluorescent proteins (FPs)) become visible. One drawback is that due to the diffraction limit (> 200 nm (Abbe 1873)) closely positioned fluorophores cannot be identified separately. The first solution offered to circumvent this problem was to actively switch closely positioned fluorophores to a different state (typically switched between an ‘on’ and ‘off’ state) (Wichmann and Hell 1994) allowing again to retrieve their individual locations. As this active switching is done at a specific known location, methods based on this principle are called coordinate-targeted SRM techniques. For chromatin studies, the most used coordinate-targeted technique is STimulated Emission Depletion (STED) microscopy.

**Fig. 1:**
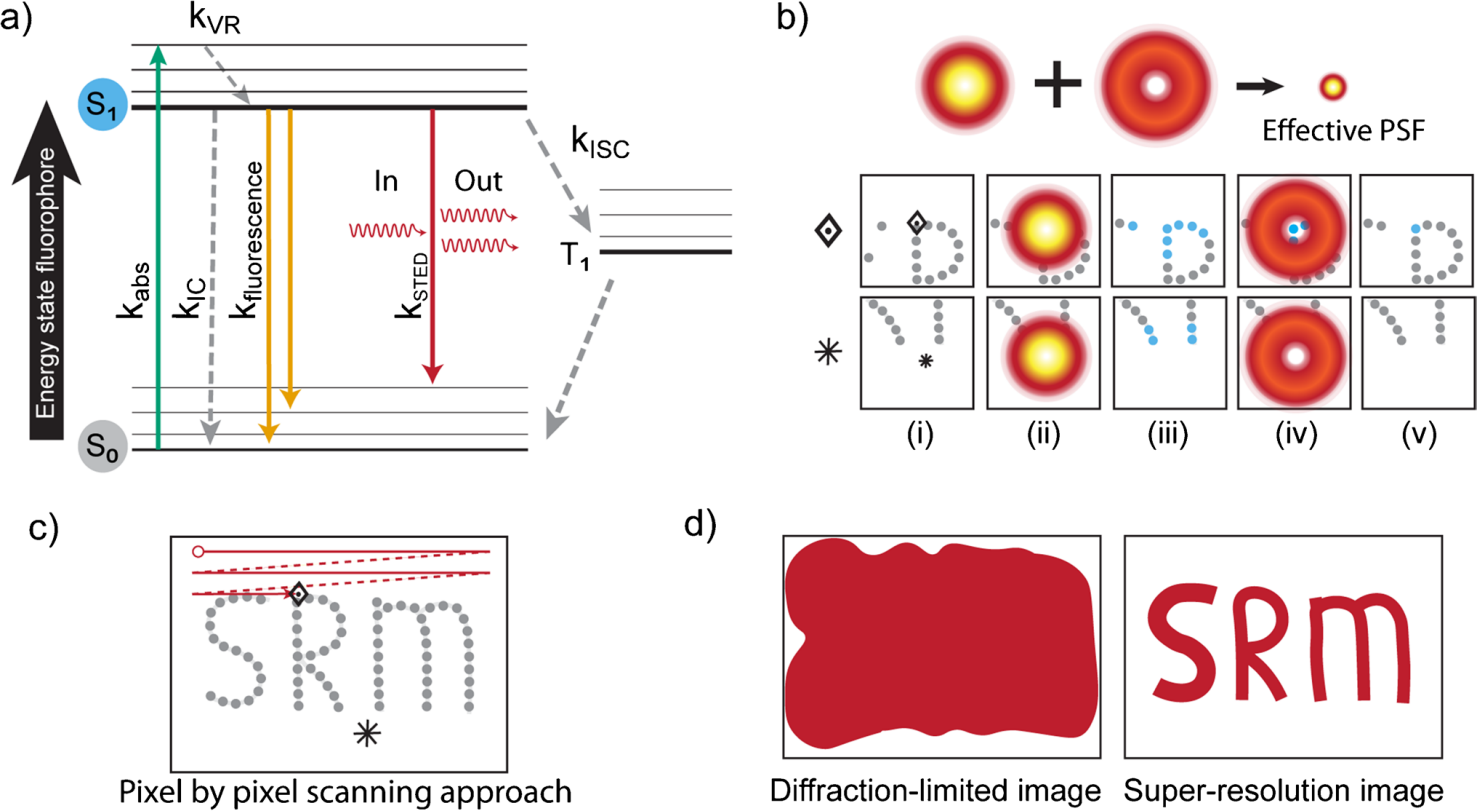
Coordinate-targeted microscopy (STED and RESOLFT). **a** Jablonski diagram showing the excitation of a fluorophore transitioning from the ground state (S0) to an excited state (S1) by absorbing an incoming photon (k_abs_). Typically, the energy is again released by spontaneous emission of a photon (k_fluorescence_). Also non-radiative processes can occur (e.g. vibrational rotations (k_VR_), internal conversion (k_IC_), or inter-system-crossing (k_ISC_) to, e.g. the triplet state (T_1_)). Fluorophores in S1 can furthermore be forced to go to the ground state through stimulated emission (k_STED_). **b** In STED microscopy, a focussed beam excites, and a (doughnut-shaped) STED beam depletes fluorophores. At each pixel (position indicated in i), fluorophores are illuminated (ii) to excite to S1 (blue in iii), and then be depleted (by doughnut beam in iv), after which the remaining excited fluorophores (blue in v), are still able to emit. **c** Pixel-by-pixel scanning results in a super-resolved image. The ‘◈’ and ‘⁕’ signs indicate the locations of the examples in **b**. **d** By scanning the entire FOV, an image with a resolution beyond the diffraction limit is acquired

To acquire a super-resolution image in STED microscopy, fluorophores are first irradiated by a focussed laser spot (~ 250 nm wide, diffraction-limited). Absorption of the incoming light brings the fluorophores into an excited state (Fig. 1a). Next, the exact same location on the sample is also irradiated by the STED beam, a red-shifted doughnut-shaped beam with no intensity in the middle of the doughnut. When this STED beam passes the already excited fluorophores, it triggers these fluorophores to release their absorbed energy by emitting light, a phenomenon called stimulated emission. Afterwards, only the non-exposed fluorophores in the centre of the doughnut can still fluoresce and be detected, reducing the effective point spread function (PSF), and thus the resolution, as illustrated in Fig. 1b. For one specific (known) location, an excite-deplete-detect cycle (Fig. 1b) thus detects only those fluorophores which were present inside the doughnut centre. Pixel-by-pixel scanning (moving either the sample or the beams, Fig. 1c) enables the construction of a super-resolved image (Fig. 1d) in real-time with a typical resolution of a few tens of nanometres.

Besides STED, other coordinate-targeted techniques include Ground State Depletion (GSD) microscopy (Bretschneider et al. 2007) and REversible Saturatable OpticaL Fluorescence Transitions (RESOLFT) microscopy (Grotjohann et al. 2011). Both GSD and RESOLFT apply switching of states, but instead of stimulated emission, GSD uses high laser powers to force fluorophores into a non-emissive state (triplet or dark state), and RESOLFT uses (low) light exposure to switch their special fluorophores (mostly FPs) between a fluorescent and a non-fluorescent configuration (often cis–trans isomerisation reactions) (Tang and Fang 2022).

#### Spatial resolution

In STED, the spatial resolution is theoretically not limited and depends on the power of the depletion laser. The higher the power, the smaller the area/volume where fluorophores are not depleted, and thus the better the resolution (the resolution scales with ${~}^{1}\!\left/ \!{~}_{\sqrt{sted\;intensity}}\right.$). Currently, the main factor limiting the resolution is the bleaching of the fluorophores upon repeated excitation-emission cycles. Typical lateral resolutions are 20–40 nm in fixed-cell imaging and 50–80 nm in live-cell imaging (Bond et al. 2022; Godin et al. 2014; Gu et al. 2020), although a resolution of 2.4 nm (Wildanger et al. 2012) has been reported for nanodiamonds. The axial resolution can be improved from the standard ~ 600 nm by shaping the depletion beam such that also fluorophores above and below the focal plane are depleted, resulting in typical axial resolutions of 80–100 nm in commercial setups (Sahl et al. 2017), but resolutions of ~ 30 nm have also been reported (Hell et al. 2015).

#### Temporal resolution

Fundamental to coordinate-targeted techniques is the requirement to know the location within the sample from where the detected light originated. Therefore, each image is constructed from individual measurements per pixel, requiring sample scanning. Hence, the acquisition time of a single image linearly depends on the number of pixels imaged, and thus on the field of view (FOV). Typically, a single super-resolved image takes about a second for FOVs below 3 × 3 µm^2^. Parallelisation of the image acquisition effectively decreases the acquisition time for both STED and RESOLFT, leading to multiple frames per second even for large image areas (50 × 50 µm^2^), and in three dimensions (Bergermann et al. 2015; Bodén et al. 2021; Chmyrov et al. 2013; Lee and Bewersdorf 2021; Masullo et al. 2018).

#### Live-cell compatibility

STED depends on high laser powers for its resolution, requiring photostable fluorophores. As a result, often organic dyes are used, which are more photostable than FPs. For live-cell imaging, a wide range of non-toxic dyes have been developed which enter cells without requiring cell lysis. Most of these excite/emit in the red, as red light has a decreased phototoxicity (lower energy per photon and less non-specific absorption by the cell) compared to more blue-shifted wavelengths. Using these labels, STED indeed showed to be live-cell compatible (Kilian et al. 2018). To further decrease the impact of light, STED offers adaptive-illumination methods. Two main strategies are Dynamic intensity MINimum (DyMIN) and Reduction of State transition Cycles (RESCue), which adapt the excitation and depletion dose to the necessity per pixel, leading to dose reductions up to 90–95% (Heine et al. 2017; Staudt et al. 2011). RESOLFT already operates at a much lower light dose as compared to STED (Chmyrov et al. 2013). Developments in RESOLFT-compatible probes led to decreased bleaching with increased contrast (Konen et al. 2021). To our knowledge, RESOLFT microscopy has not been applied to study chromatin yet, but these lower light doses might make it a versatile tool to investigate chromatin dynamics in living cells in the near future.

#### Multi-colour imaging

To unravel cellular mechanisms, it is often crucial to visualise more than one protein at a time. The large variety of dyes makes dual-colour STED microscopy straightforward. In combination with large-Stokes-shift dyes (Sednev et al. 2015), up to three colours can be imaged simultaneously. Additionally, lifetime multiplexing could offer options even beyond three colours with the same depletion dye, and thus without chromatic aberrations (Frei et al. 2022).

#### Post-processing

A major advantage of coordinate-targeted techniques is that they directly determine the signal per location/per pixel, omitting the need for post-processing. Samples thus can be evaluated immediately, which in principle also allows for high-throughput measurements (Alvelid et al. 2022; Mol and Vlijm 2022).

## Stochastic localisation super-resolution microscopy

### Technique

**Fig. 2:**
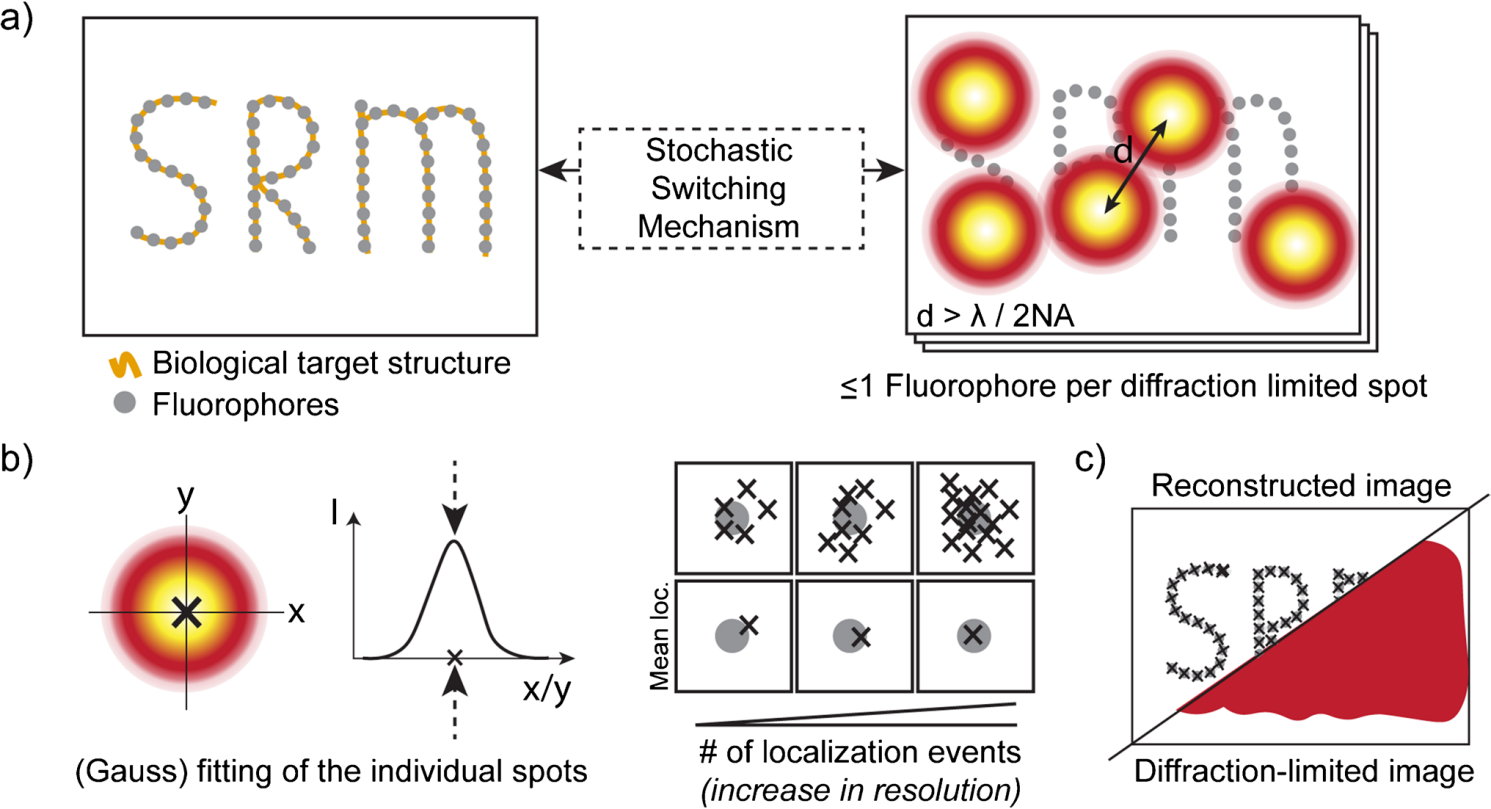
Single-molecule localisation microscopy (SMLM). **a** In SMLM, the target is labelled with fluorophores which stochastically switch between an ‘off’ and ‘on’ state. Each detected PSF of a fluorophore should be spatially separated by at least the diffraction limit. Stochastic switching for STORM and dSTORM/GSDIM is often referred to as blinking, while PALM fluorophores in most cases do not blink but instead give a burst of photons upon stochastic ‘on’ switching. **b** By applying a fit (for instance gauss fit) to the PSF of the fluorophore, the centre position can be determined. The more localisations (photons) per fluorophore, the better the localisation accuracy. **c** By combining all localisation events, a SRM image can be reconstructed

Stochastic-based localisation super-resolution microscopy (often referred to as single-molecule localisation microscopy (SMLM)) is camera-based and, unlike coordinate-targeted SRM, does not require sample scanning. In SMLM, fluorophores which are in closer proximity than the diffraction limit are detected separately by stochastically switching them between an emissive ‘on’ and ‘off’ state. The experimental conditions are tuned such that at most one fluorophore per diffraction-limited area is emitting (Fig. 2a). For each detection event, the centre of the spot (point spread function (PSF)) is determined (Fig. 2b). The most general approach for determining the centre is a gauss fit (Stallinga et al. 2010), although there are more enhanced methods (Babcock and Zhuang 2017; Li et al. 2022b; Nehme et al. 2021). By summing many frames (typically ~ 50,000), the entire population of fluorescent molecules can be localised (Fig. 2c). In SMLM, the resolution thus scales with the number of collected frames, coupling the temporal resolution (minutes to hours per image (Heydarian et al. 2018)) to the lateral resolution (typically 20–50 nm (Bond et al. 2022)).

There are several SMLM techniques which differ in how they stochastically switch (subsets of) fluorophores between ‘off’ and ‘on’ states. Here, we will discuss: (1) fluorescently Photo-Activatable Light Microscopy ((f)PALM) (Betzig et al. 2006; Hess et al. 2006), (2) STochastic Optical Reconstruction Microscopy (STORM) (Rust et al. 2006), (3) direct-STORM (dSTORM) (Heilemann et al. 2008) or the technical similar Ground State Depletion microscopy followed by Individual Molecule return (GSDIM) (Bretschneider et al. 2007; Fölling et al. 2008), (4) Point Accumulation for Imaging in Nano Topography (PAINT) (Sharonov and Hochstrasser 2006), and (5) DNA-PAINT (Jungmann et al. 2010).

**Fig. 3:**
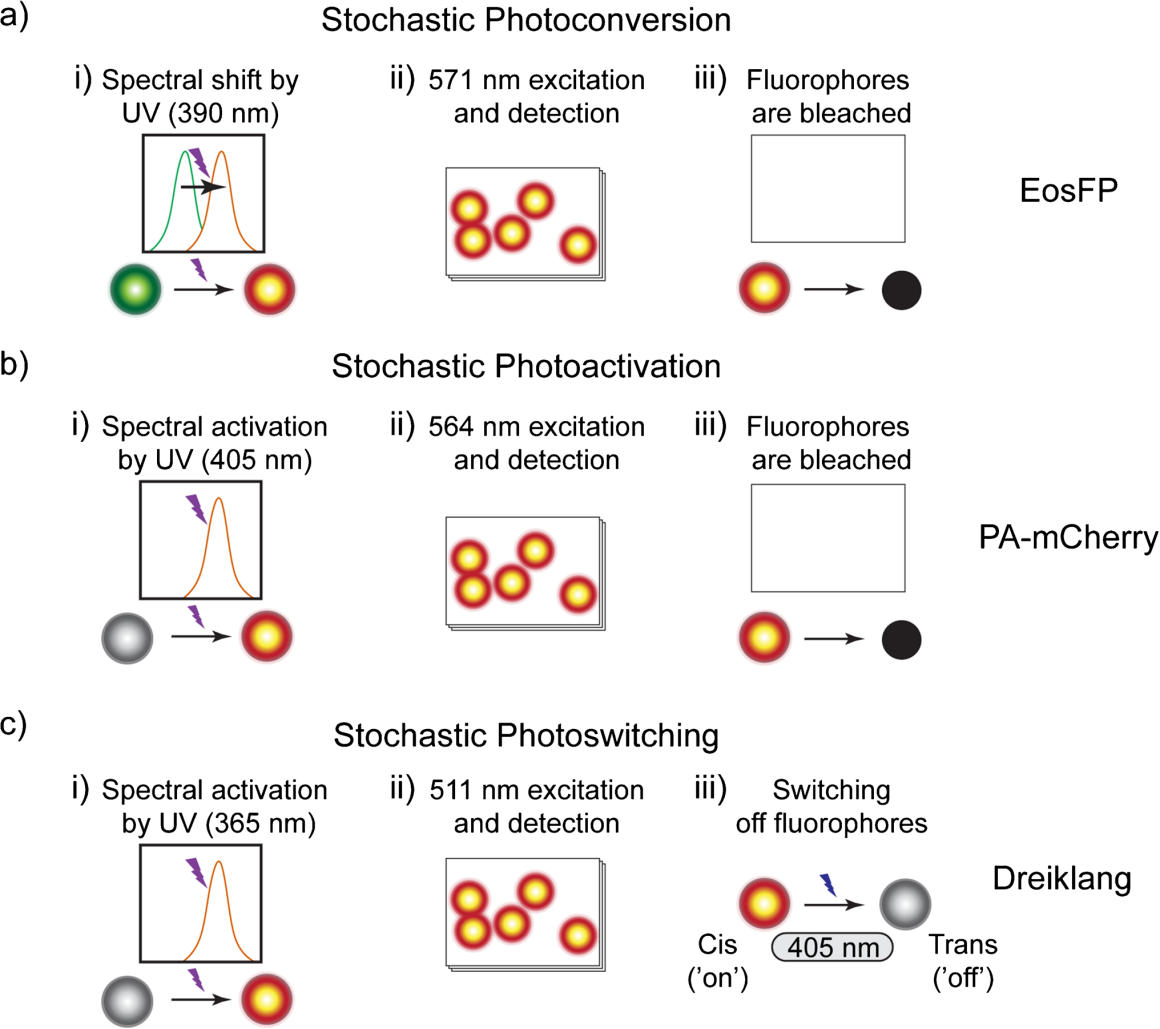
Switching mechanisms for (f)PALM, using EosFP, PA-mCherry, and Dreiklang as examples. **a** Photoconversion: irradiation (at 405 nm for EosFP) induces a shift in the spectral absorption of a subset of the fluorophores. By consecutive irradiation at this new absorption wavelength (e.g. 540 nm for EosFP), only this subset is allowed to fluoresce. These are irradiated and detected until they are all bleached. **b** Photoactivation: similar to photoconversion (see **a**), but the initial form does not exhibit fluorescent properties. **c** Photoswitching: fluorophores (e.g. Dreiklang) can reversibly switch between a fluorescent and non-fluorescent state upon irradiation

PALM (TIRF-based microscopy) and (f)PALM (confocal microscope) both use FPs. These FPs can have three different switching mechanisms, namely photoconversion (Fig. 3a), photoactivation (Fig. 3b), or photoswitching (Fig. 3c). Here, we will give concrete examples of these mechanisms. Photoconvertible FPs (EosFP (Betzig et al. 2006)) are fluorescent, but upon irradiation with a specific wavelength (390 nm for EosFP), they show an irreversible shift in the excitation spectrum (from 506 to 571 nm for EosFP (Fig. 3a)). A short pulse with the activation wavelength stochastically activates a subset of (spatially separated) FPs. Consecutive irradiation at the new absorption maximum (571 nm for EosFP) leads to a burst of photons until those fluorophores are bleached, and a new subset can be activated. Photoactivatable FPs, like PA-mCherry (Subach et al. 2009), are non-fluorescent until activated by light (405 nm for PA-mCherry). In their activated state, they emit upon irradiation with another wavelength (564 nm for PA-mCherry) until they are bleached (Fig. 3b). The burst of photons from photoconvertible and photoactivatable FPs allows for the tracking of fast dynamics, e.g. live-cell single-particle tracking (sptPALM) (Manley et al. 2008). Alternatively, FPs can be used which can reversibly switch between a fluorescent ‘on’ and ‘off’ state upon illumination with two different wavelengths (e.g. Dreiklang (Brakemann et al. 2011), Fig. 3c).

In STORM, an activator-reporter dye pair is used as label. Here, the activator-parts are stochastically activated by laser irradiation, upon which energy transfers enable their reporter dye to emit. The advantage of this approach is its applicability to multi-colour imaging. Often, multi-colour SMLM approaches suffer from chromatic aberrations. Combining spectrally different activators with the same reporter dye solves this issue (Bates et al. 2007), but these different structures have to be recorded consecutively, requiring even better drift corrections (Lelek et al. 2021). A disadvantage of this activator-reporter dyes is the often significant size (~ 30 nm), which typically limits the resolution (Bates et al. 2007). Recent optimisations towards smaller labels increased the labelling density and improved the resolution (Chen et al. 2016).

**Fig. 4:**
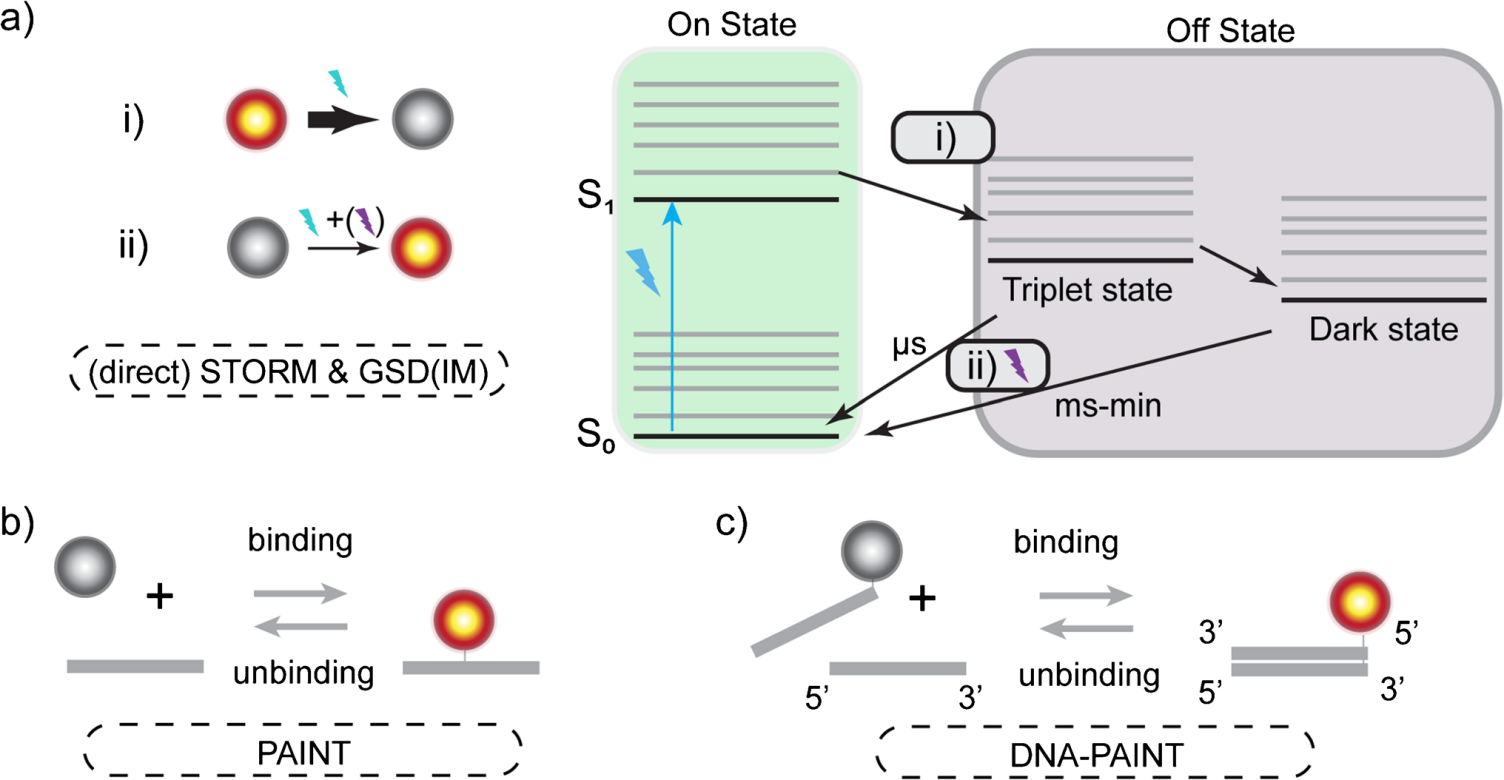
GSDIM/dSTORM and (DNA-)PAINT. **a** In GSDIM/dSTORM (i), all fluorophores are pushed into (long-lived and non-emissive) triplet or dark states, typically by applying high laser powers. When all fluorophores are ‘off’, the laser power is significantly reduced (~ 10 times). (ii) When fluorophores return to the ground state (in a stochastic manner), they can absorb and emit again, which will be detected. This cycling between on and off states (blinking) is repeated until the fluorophore is bleached. Specialised imaging buffers and irradiation by UV light are sometimes used to increase/adapt the rate at which fluorophores in the ‘off’ state (dark/triplet state) return to the ground state. **b** PAINT and **c** DNA-PAINT both use exchangeable and fluorogenic (only fluorescent upon binding) probes. The binding kinetics dictate the stochastic detection of the molecules

In GSDIM/dSTORM, a different mechanism for stochastic switching is used. Here, first all molecules are forced into an off-state (metastable triplet states or dark-states) by applying a high-intensity laser (Fig. 4a). Next, the laser power is typically tuned down by a factor of ten. The fluorophores reside in this off-state for µs up to minutes, after which they stochastically return to the ground state, where they can absorb and emit again (on-state). The constantly present (low) excitation laser then enables these on-state molecules to fluoresce, until they return to the off-state. This process is often referred to as blinking. For GSDIM/dSTORM, typically organic dyes are used as fluorophore, as in general FPs are not stable enough. To further reduce the photobleaching, specialised imaging buffers are added, which unfortunately often contain cytotoxic components, making live-cell imaging more challenging.

Where previously discussed SMLM approaches mainly use covalently bound dyes, PAINT (Fig. 4b) and DNA-PAINT (Fig. 4c) rely on the exchange of fluorescent probes. Here, the probe binding and unbinding kinetics dictate the on- and off-switching rates, as the probes only fluoresce upon binding. One important advantage is that photobleaching no longer poses a problem as bleached fluorophores can be replaced. However, the sample stabilisation and drift correction become more challenging, as the binding-dependent switching typically results in longer acquisition times (Heydarian et al. 2018) compared to other SMLM techniques.

#### Spatial resolution

Similar to coordinate-based approaches, the resolution is theoretically not limited. In SMLM, the resolution scales with ${~}^{1}\!\left/ \!{~}_{\sqrt{detected\;photons\;per\;fluorophore}}\right.$, which couples the temporal resolution to the spatial resolution. Currently, the main factors limiting the spatial resolution are fluorophore bleaching, the size and position of the fluorophore, and drift. In practice, the lateral resolution of SMLM is typically 20–50 nm (Bond et al. 2022). Besides the lateral resolution, the axial resolution can also be improved to allow for 3D imaging (Lelek et al. 2021).

#### Temporal resolution

The temporal resolution in SMLM is generally minutes to hours per image (Heydarian et al. 2018); however, a temporal resolution of seconds was achieved using recent sCMOS technology (Ma and Liu 2020). The obtained temporal resolution depends on the camera frame rate, the rate at which the fluorophores stochastically switch, the (aimed) spatial resolution, and the labelling density. To control the switching rate, one can vary the laser power density (PALM, STORM, dSTORM/GSDIM (Lelek et al. 2021; Tang and Fang 2022)), tune the blinking-buffer (dSTORM/GSDIM (Nahidiazar et al. 2016)), tune the label properties (Lardon et al. 2021) and adapt the label concentration (PAINT/DNA-PAINT (Schueder et al. 2017)), or the oligo design (DNA-Paint (Beliveau et al. 2017)).

#### Live-cell compatibility

PALM and PAINT are most compatible with live-cell imaging. Other SMLM techniques are more challenging for live-cell imaging as often toxic imaging buffers are required to tune the blinking properties of the fluorophore. However, some live-cell protocols have been developed (Jones et al. 2011; Morozumi et al. 2020; Oi et al. 2020; Teng et al. 2016).

#### Multi-colour imaging

Multi-colour imaging with blinking-based methods is challenging as it requires buffer conditions in which all fluorophores optimally blink. The OxEA imaging buffer in combination with Alexa dyes was found to allow up to three colour imaging simultaneously (Nahidiazar et al. 2016). Another approach for three colour imaging is through the use of three activator-reporter dye pairs that can be activated separately but which have the same reporter dye, ruling out chromatic aberrations (Testa et al. 2010). Very recently a method called excitation-resolved STORM even enabled four colour imaging (Wu et al. 2023). A complicating factor for multi-colour SMLM is the need for specialised fitting algorithms (Kim et al. 2013; Li et al. 2022b; Shechtman et al. 2016). PAINT approaches however (although bound to fixed samples) can label with numerous probes sequentially (Ma and Liu 2020).

#### Post-processing

Unlike coordinate-targeted techniques (which directly acquire their super-resolution image), SMLM requires computationally intensive pre- and post-processing, although efforts are made to reduce the computational power required (Babcock and Zhuang 2017). Examples of pre-processing steps include applying fitting algorithms and correcting for camera artefacts, drift and multiple blinking (Lelek et al. 2021). For post-processing many different methods are available to segment and quantify the composition of nano-clusters (Bond et al. 2022).

## SRM-compatible labels for chromatin research

Important for microscopy studies on chromatin is the possibility to label the DNA, chromatin-associated proteins, and specific epigenetic states. Here, we will review the current labelling strategies applicable to SRM. We refer to other reviews for a more extensive discussion on other labelling aspects and a more elaborate list of fluorophores, including small- (Grimm and Lavis 2022), photoswitchable- (Chozinski et al. 2014; Endesfelder et al. 2011), organic- (Dempsey et al. 2011), live-cell-compatible- (van de Linde et al. 2012), STED-specific- (Jeong and Kim 2022; Kostiuk et al. 2019), and epigenetic-state-probing (Stepanov et al. 2022) fluorophores.

### DNA labelling

There are various strategies to label DNA with fluorescent markers. One method is through the introduction of thymidine analogues such as 5-ethynyl-2′-deoxyuridine (EdU), which is inserted into the DNA upon DNA synthesis. EdU itself is non-fluorescent and has to be conjugated with a dye, such as azide-CF568 or azide-Alexa 647 (Xu and Liu 2021), where F-ara-EdU was found to be least disruptive (Hao et al. 2021). The click chemistry reaction for conjugation requires fixation of the cell. Alternatively, the DNA backbone can also be labelled in living cells by introducing already fluorescent nucleotides (e.g. ATTO 633-dUTP, ATTO 565-dUTP) through scratch replication labelling (SRL) (Schermelleh et al. 2001). Gentle permeabilisation to aid the take-up was found to be beneficial (Xiang et al. 2018). A second strategy is through fluorescently labelled oligonucleotides (OligoPaints). The standard method here is to fix cells and denature the DNA to allow for hybridisation with fluorescently labelled oligonucleotides (e.g. ATTO 655, ATTO 565, AlexaFluor 488), enabling to detect specific sequences (Beliveau et al. 2015; Beliveau et al. 2017). This method has been successfully applied using SMLM to visualise chromatin from single loops up to the 3D chromosome (Nguyen et al. 2020; Parteka-Tojek et al. 2022). One note for careful consideration is that this method might induce changes in the chromatin structure during the necessary DNA denaturation (Hao et al. 2021). Alternatively, genetic engineering approaches omit the need for DNA denaturation and enable live cell imaging of repetitive genomic loci (Chen et al. 2013) and more recently also nonrepetitive sequences (Clow et al. 2022; Lyu et al. 2022). A third labelling strategy is to label the native DNA with groove-binding or DNA intercalating dyes, such as DAPI, Hoechst (Bucevičius et al. 2018), and cyanine-based dyes (e.g. PicoGreen (Benke and Manley 2012) and TOTO®-3 (Xu et al. 2020)). DAPI is in general not used for SRM applications as Hoechst and cyanine-based dyes have better spectral properties and are less cytotoxic (Bucevičius et al. 2018). Various Hoechst-derived dyes are optimised for live-cell SRM with a reduced cytotoxicity, optimised cell permeability, and increased fluorogenicity (only fluorescent upon specific binding), including JF_646_-Hoechst (Grimm et al. 2017), SiR-Hoechst, Cy5-Hoechst, HoeSR Rhodamine-Hoechst isomers, 5-HMSiR-Hoechst (Bucevičius et al. 2020), 4-TMR-Hoechst, and 4-580CP-Hoechst. Which label is most suitable depends on the application. For example, 4-TMR-Hoechst and 4-580CP-Hoechst are well suited for live-cell imaging due to their increased biocompatibility, requiring a 100-fold lower concentration compared to Hoechst-based predecessors (Bucevičius et al. 2020). Also, the highly fluorogenic 5-HMSiR-Hoechst is a good candidate for SRM live-cell imaging (both STED and SMLM), as it has a good DNA binding constant, low toxicity, and a 400-fold increase in fluorescence upon binding. A fourth approach is to label the DNA indirectly through the histone proteins (see ‘Chromatin-associated protein labelling’ section). The increased distance between the fluorophore and the DNA backbone could slightly reduce the localisation precision, but in live-cell imaging this distance could prevent/reduce imaging induced DNA damage.

### Chromatin-associated protein labelling

Chromatin-associated or interacting proteins can be labelled through immunofluorescence labelling (IF) (e.g. histone H2B (Ricci et al. 2015)), endogenous or exogenous expression of a fluorescent protein (Wu et al. 2019), or by the expression of a self-labelling enzymatic tag at the target molecule which allows live-cell imaging (e.g. histones H2B-Halo/H2B-SNAP (Nozaki et al. 2017; Ricci et al. 2015)). In immuno-fluorescence approaches, the selected secondary antibody should be adequate for the SRM technique. For STED microscopy, commercial secondary antibodies (e.g. from Abberior) allow for straightforward two-colour (STAR 580 and STAR 635) or even three-colour aberration-free imaging when utilising a long Stokes shift dye (STAR 460L). Other types of STED-compatible dyes include rhodamines (such as SiR (Lukinavičius et al. 2013), JF585 (Grimm et al. 2017), TAMRA-6 and MaP probes (Wang et al. 2019), Atto 647N and N-cyanorhodamines (Heynck et al. 2022)), Alexa Fluor 595, long Stokes shift dyes such as YL578 (Jiang et al. 2022), and photoactivatable SiR or xanthones dyes (Lincoln et al. 2022; Weber et al. 2021). The most frequently used immunofluorescent label in STORM microscopy is Alexa Fluor 647 (many distributors), in combination with other Alexa Fluor dyes for multi-colour imaging. Also, CAGED dyes (Belov et al. 2009) are a good solution for SMLM, as they are non-fluorescent in their caged form and become uncaged and fluorescent upon UV irradiation. The previously mentioned rhodamines are also compatible with SMLM as their blinking properties can be tuned by modifying their spirocyclisation (Lardon et al. 2021).

Instead of IF, self-labelling can be achieved through enzyme tags (like Halo, SNAP, and CLIP) engineered to be expressed by the target molecule, in combination with fluorescent dyes which bind to these tags (e.g. SiR-Halo, Janelia Fluor® HaloTag® Ligands, YL578-Halo, 580CP-Halo, CPY-Halo or SNAP-Cell® 647-SiR). A major advantage is that this method allows for live-cell imaging (Butkevich et al. 2017; Grimm et al. 2017) and pulse-chase experiments (Gautier et al. 2008; Yamaguchi et al. 2009). Images furthermore show significantly less background compared to IF, due to the fluorogenic properties of the dyes (Wang et al. 2019), and the tendency of cells (in live-cell labelling) to remove unbound dyes. Alternatively, in bacteria, Chemogenetic Tags with Probe Exchange (CPTEs) (Iyer et al. 2021) in conjunction with various fluorophores can be used for long-term imaging. More recently, exchangeable HaloTags ligands have been developed. By allowing the replacement of photobleached fluorophores, better resolutions and/or long-term imaging can be achieved (Kompa et al. 2023).

### Assessing chromatin compaction and epigenetics

Chromatin compaction can be assessed by analysing the DNA density directly (Martin et al. 2021), or by using FRET-FISH probes (Mota et al. 2022). Often also more indirect methods are applied, using transcription activity (Martin et al. 2021) or epigenetic histone modifications (Stepanov et al. 2022) as indicators. Alternatively, an assay that probes transposase-accessible chromatin (ATAC) can be used, as the genome accessibility relates to its compaction (Xie et al. 2020).

## Important considerations for SRM

### Technique selection

Which SRM technique is most suitable depends on the research question. To aid the selection process, Table 1 compares important aspects of the SRM techniques included in this review.

**Table 1:**
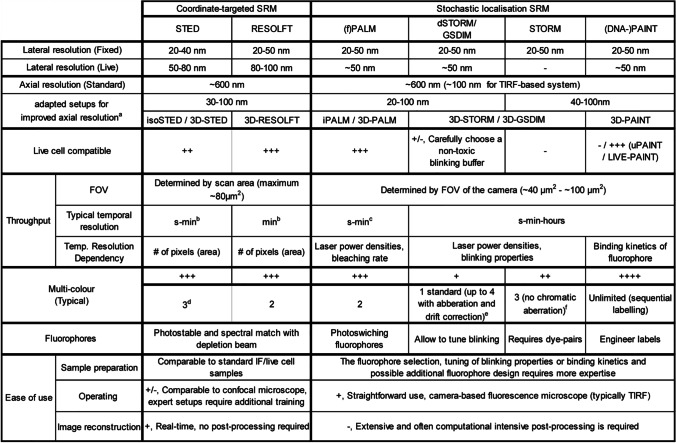
Overview of the different SRM techniques discussed. The reported numbers are taken from Bond et al. (2022), Godin et al. (2014), Sahl et al. (2017), and Sahl and Hell (2019)

^a^Obtained axial resolution depends on the axial improvement method (Sahl and Hell 2019). ^b^The temporal resolution can be significantly improved through parallelised acquisition (Bergermann et al. 2015; Bodén et al. 2021; Chmyrov et al. 2013; Lee and Bewersdorf 2021; Masullo et al. 2018); however, these setups are not (yet) commercially available. ^c^sptPALM can provide dynamic information of single molecules at millisecond timescale (Manley et al. 2008). ^d^Three colours can be obtained with commercially available fluorophores of which one should be a long Stokes shift dye (Sednev et al. 2015), for additional colours lifetime multiplexing could be considered (Frei et al. 2022). ^e^Possibility of imaging three or more colours, recently even four colours (Testa et al. 2010; Wu et al. 2023). ^f^This multi-colour approach is prone to crosstalk between colours and needs correcting (Bates et al. 2007)

### Sample optimisation

**Fig. 5:**
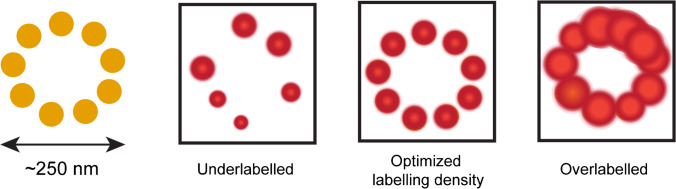
The degree of labelling influences the image quality (Lau et al. 2012)

With the increased resolution of SRM, the label size and degree of labelling significantly influence the final image. Therefore, it is recommended to use small labels for SRM (FPs, nanobodies, aptamers, affimers, enzymatic tags or unnatural amino acids) (Grimm and Lavis 2022; Sahl et al. 2017). Additionally, the labelling density should be optimised through a titration of the label concentration and/or incubation time as under-labelling results in gaps and over-labelling in blurring of the structure (Fig. 5) (Lau et al. 2012). The photon budget and signal-to-noise ratio can be maximised by (i) minimising the contact of the fluorophore with water (Maillard et al. 2021); (ii) properly storing the fluorophore; (iii) adding photoprotective agents to the mounting media for fixed samples or (when non-toxic) to the medium in live-cell applications (Gong et al. 2019; Noa et al. 2021); (iv) applying adaptive-illumination strategies; (v) optimising laser-excitation powers, preventing unnecessary high bleaching; and (vi) using phenol-red-free media when imaging in the red. Other sample optimisations not specific to SRM, such as labelling artefacts, are reviewed by, e.g. Sograte-Idrissi et al. (2020).

### Multi-colour imaging

To enable bleed-through corrections in multi-colour imaging, the individual colours should be measured in single-colour samples using the final excitation and detection configurations. Additionally, the more red-shifted label is typically to a higher degree also excited by the more blue-shifted excitation laser than vice versa. Therefore, it is recommended to label the least abundant protein with the more red-shifted fluorophore.

### Live-cell imaging

**Fig. 6:**
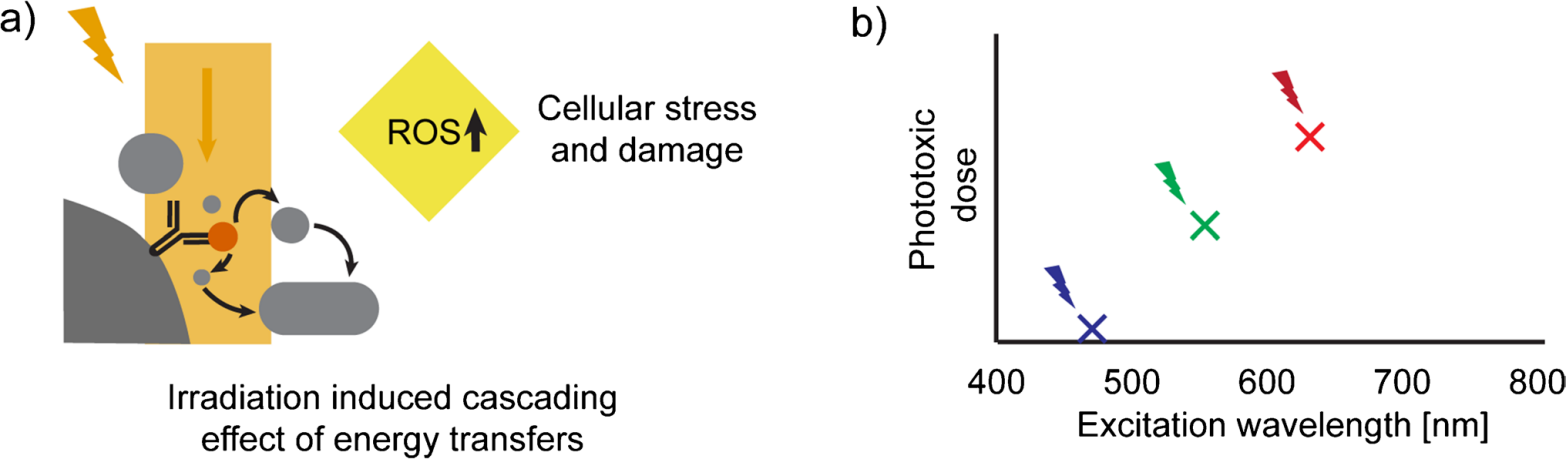
Live-cell-labelling considerations. **a** Photons can cause a cascade of energy transfer events, resulting in an increased ROS concentration, inducing cellular stress and damage (Ojha and Ojha 2021). **b** Cells absorb less light towards the (far-)red, reducing the phototoxicity by (far-)red light (Emon et al. 2021)

The chromatin structure and dynamics might be perturbed by the presence of a fluorescent label or by laser irradiation. When light interacts with cells (get absorbed), a cascade of energy transfer events can either change molecular structures directly, or it can lead to increased concentrations of reactive oxygen species (ROS), which can induce cellular stress, alter cellular processes, or lead to (DNA) damage (Ojha and Ojha 2021) (Fig. 6a). To minimise this effect, wavelengths in the (far-)red should be chosen as these are less absorbed than more blue wavelengths (Arai et al. 2015). For example, blue light is phototoxic at a 20-fold lower dose than red light (Emon et al. 2021; Waldchen et al. 2015) (Fig. 6b). Additionally, light-dose-reducing strategies are strongly recommended (e.g. Dymin/Rescue for STED (Heine et al. 2017; Staudt et al. 2011)). Another source for (increased) phototoxicity might be the fluorophore itself (Hofmann and Weber 2021; Kowalska et al. 2021), which sometimes can be solved by using a variant of the fluorophore with a different charge or binding kinetics (Bucevičius et al. 2019; Hao et al. 2021; Kähärä et al. 2022).

## Chromatin architecture unveiled by SRM

SRM techniques have been successfully utilised to unveil the role of the spatial organisation of chromatin at the sub-diffraction-limited length scale, leading to a better understanding of the functioning of DNA, histone modifications, and other chromatin-associated proteins. A major benefit of SRM techniques is their ability to obtain spatial information at near-biomolecular resolution in the native environment within individual cells, allowing to observe subpopulations or obtain data from a specific cell-cycle state (e.g. cell division). Also difficult to obtain using other approaches, but shown by SRM, are investigations on how the epigenetic state influences chromatin folding (3D-STORM) (Boettiger et al. 2016), and mapping of the methylation content of individual telomeres, ribosomal genes or centromeres (STED, STORM) (Franek et al. 2021). Additionally, chromatin (re)organisation as a result of diseases or external mechanical or chemical cues can be probed (STORM) (Heo et al. 2022; Xu et al. 2022).

### Chromatin compartmentalisation

Chromatin interaction maps (3C-based methods) have shown in population-averaged data that chromatin compartmentalises into megabase-scaled topologically associated domains (TADs, Hi-C) (Dixon et al. 2012), kilobase scaled loops, and even smaller sub-loops (Hi-C) (Rao et al. 2014). These TADs have increased chromatin interactions and a higher density of promotors and enhancers within the domain, and reduced interactions at their borders (Hi-C) (Dixon et al. 2012). SRM imaging has demonstrated that chromatin indeed compartmentalises, as shown by clusters of dense DNA or DNA interacting proteins. Examples of these clusters include condensed mitotic chromosome structures (PALM, fixed, ~ 70 nm) (Matsuda et al. 2010); nuclear nanostructures (2D localisation light microscopy, fixed, < 100 nm) (Bohn et al. 2010); nucleosome clutches or compacted domains (PALM and STORM, live and fixed, ~ 160 nm) (Nozaki et al. 2017; Ricci et al. 2015); TAD-like domains (Multiplexed 3D-STORM FISH compared with Hi-C, fixed, ~ 300 nm) (Bintu et al. 2018); elongated-chromatin ‘blobs’ (Deep-PALM, live, < 100 nm) (Barth et al. 2020); chromocenters (STED, fixed, 90–150 nm) (Erdel et al. 2020); packing domains (nano-ChIA: multimodal platform including STORM, fixed, 200 nm) (Li et al. 2021a); nanodomains (STORM, fixed, 115–160 nm) (Xu et al. 2022); and replication domains (STORM, live, 150 nm) (Xiang et al. 2018). Factors of influence on the size estimation of these domains are (i) the type of cells used, as the domain size could be affected by disease or pluripotency (STORM, live and fixed) (Ricci et al. 2015; Xu et al. 2022); (ii) the acquisition time, as sub-second remodelling dynamics could lead to an overestimation of the size after prolonged imaging (Deep-PALM, live) (Barth et al. 2020); (iii) sample preparations and setup limitations; and lastly, (iv) an intrinsic heterogeneity in size, morphology, and distribution even within the same cell type (STORM and Multiplexed 3D-STORM FISH, live and fixed) (Nozaki et al. 2017; Su et al. 2020a).

### Nucleosome clutches

Using STORM (live and fixed) microscopy, clusters of nucleosomes (clutches) were observed (Ricci et al. 2015). The clutch size was shown to be highly heterogeneous and the median increased upon cell differentiation (STORM, live and fixed) (Nozaki et al. 2017; Ricci et al. 2015). Smaller clutches typically had a higher degree of acetylation (Ricci et al. 2015) and acetylated histone tails led to more loosely packed DNA and a decreased nucleosome occupancy. This effect was enhanced in nucleosome-rich areas (STORM-PAINT, fixed) (Otterstrom et al. 2019). Comparing clutches with similar acetylation densities revealed that the clutch size did not influence the DNA density. Small nucleosome clutches (30–50 nm) were found to be typically more transcriptionally active as elongated clusters of RNAP II with nascent RNA associated with these clutches (STORM and STORM-PAINT, fixed) (Castells-Garcia et al. 2022). Investigating the effect of hypo-osmotic and hypertonic treatment on clutch size resulted in respectively reduced and increased DNA condensations (STORM and STED, live and fixed) (Nozaki et al. 2017; Olins et al. 2020). SRM thus revealed how nucleosomes are clustered in clutches of various sizes, with acetylated histone modifications preferentially located in small, transcriptionally active clutches.

### DNA loop formation

Within TADs, chromatin arranges into loops at the kilobase length scale (Hi-C) (Rao et al. 2014). One proposed mechanism for loop formation is the loop extrusion model (Alipour and Marko 2012). In this model, cohesin is the loop-extruding factor, leading to a continuously growing loop. The loop stops growing when it encounters an insulating boundary element, such as the CCCTC-binding factor (CTCF) (Hi-C) (Fudenberg et al. 2016). Hi-C and Chromosome Conformation Capture Carbon-Copy (5C) interaction maps were able to show that the formation and properties of TADs are affected by CTCF, cohesin, and RNA polymerase II (RNAP II) (Nora et al. 2017; Rao et al. 2014; Rao et al. 2017). SRM enables to investigate the physical size and localisation of chromatin (sub)structures and can help to reveal how the spatial organisation is affected by these factors.

#### CTCF transiently interacts with cohesin to form loops

Live-cell 3D-PALM revealed that TAD loops are highly dynamic, and only during 8% of the time are found in a CTCF-CTCF looped state (Gabriele et al. 2022). Similarly, iPALM visualisation of 13-kb-sized loops showed that they have a high heterogeneity in their appearance over time. It was suggested that this might be a result of cohesin-mediated extrusions and other factors such as nucleosome stacking (Parteka-Tojek et al. 2022). These recent SRM findings complement findings using other techniques, which suggest that CTCF undergoes transient interactions with cohesin (single-molecule in vitro assay; X-ray crystallography; Capture-C, Hi-C, 4C-seq and integration site mapping sequencing) (Davidson et al. 2022; Li et al. 2020; Mach et al. 2022).

#### The role of cohesin in loop formation

In bulk Hi-C studies, the depletion of cohesin appeared to result in a disappearance of TADs (Rao et al. 2017; Schwarzer et al. 2017). Single-cell Hi-C, however, revealed that TADs were maintained upon cohesion depletion, although the domain boundaries did no longer exhibit a preferential position towards CTCF/cohesin sites (Bintu et al. 2018). Imaging these domains individually using SRM revealed that their physical size increased and their smoothness decreased upon cohesion depletion (3D-PALM and ZOLA-3D) (Gabriele et al. 2022; Hao et al. 2021).

#### The role of RNAP II in loop formation

RNAP II is typically located within TADs at the ‘tops’ of the loops formed by cohesin (STORM/DNA-PAINT, fixed) (Neguembor et al. 2021). RNAP II transcription modulates the cohesin distribution and mobility by altering the negative supercoiling. Furthermore, transcription decreases the clustering of CTCF (STED, live) (Gu et al. 2020). These studies reveal that transcription by RNAP II affects loop dynamics.

#### The role of other loop interacting factors

Chromatin organisation is also strongly dependent on cohesin interacting factors such as WAPL (responsible for cohesin unloading). Using Hi-C, it was shown that WAPL depletion (leading to cohesin overloading) leads to an increased loop size and the vermicellification of chromatin (Bintu et al. 2018; Neguembor et al. 2021; Wutz et al. 2017). STORM imaging in WAPL depleted cells revealed that RNAP II and topoisomerase activity are responsible for this vermicellification and that inhibition of either RNAP II or topoisomerases impairs loop extrusion (STORM/DNA-PAINT, fixed) (Neguembor et al. 2021). Taken together, SRM on loop structures showed that (i) cohesin mediates loop formation; (ii) these loops are highly dynamic, among others due to transient interactions between cohesin and CTCF; and (iii) the loop structure is affected by the supercoiling density resulting from RNAP II transcription.

### The spatial organisation of transcription factories

Transcription factories, also called transcriptionally active pockets, are transcription-rich chromatin regions marked by clusters of RNAP II, transcription factors, and RNA transcripts. These factories have sizes below the diffraction limit (40–200 nm) (PALM, STORM, STED, live and fixed) (Castells-Garcia et al. 2022; Cisse et al. 2013; Gu et al. 2020). The RNAP II clustering in transcription factories is rather short-lived, with an initial reported average lifetime of ~ 5 s (PALM, live) (Cisse et al. 2013). Later, endogenous studies (PALM, live) reported similar lifetimes of ~ 8 s for differentiated cell lines (Cho et al. 2016) and ~ 11 s for an embryonic mouse stem cell line (tcPALM, live) (Cho et al. 2018). Upon differentiation of the cells, the size and number of these stable clusters decreased (Cho et al. 2018). Additionally, external stimuli can (indirectly) induce changes in the RNAP II dynamics. For example, nuclear actin has been demonstrated to enhance RNAP II clustering (PALM and STORM, live and fixed) (Wei et al. 2020). This dynamic and transient RNAP II localisation, depending on many factors including the pluripotency of the cell, could explain the broad distribution of the total number of factories per cell in earlier studies.

### The spatial organisation of replication domains

In S-phase DNA replication is initiated at replication origins. Where bacteria only have a single replication origin, mammalian cells have many. Using EM, ~ 200 up to ~ 1000 replication domains (RDs) have been reported (Hozák et al. 1993; Koberna et al. 2005), all too few to be single replicons (locations of replication) based on the replication speed and typical S-phase duration in mammalian cells. Hence, the presence of multiple replicons within a single RD (Rivera-Mulia and Gilbert 2016) was suggested. Early SRM (STED) allowed to visualise these RDs in their cellular environment revealing a much higher number of RDs per cell (~ 5000) with a size of about ~ 150 nm (fixed) (Cseresnyes et al. 2009). With an estimated 25 k–30 k number of replicons per cell, still about five replicons were expected to be present inside each RD. Later, with an improved resolution (~ 20 nm, STORM) it could be shown that these 150–160-nm RDs comprise of four replication forks (four replicons) each spaced 63 nm apart (fixed) (Xiang et al. 2018). Nucleosome clutches colocalised with these RDs and moved coherently (PALM, live) (Nozaki et al. 2017; Xiang et al. 2018). Active RDs were furthermore shown to have specific locations during S-phase, under the influence of CTCF (CTCF depletion led to a random distribution) (STORM, fixed) (Li et al. 2021b; Su et al. 2020b). Where in early S-phase active RDs were found more centrally in the nucleus and comprised of four replicons on average, upon progression to mid and late S-phase the RD size was found to increase, containing respectively 7 and 10 replicons per RD (STORM, fixed) (Su et al. 2020b).

## Conclusion and outlook

Here, we presented an overview of SRM, how best to apply it to chromatin studies, and through a selection of examples the versatility of SRM for chromatin studies was illustrated. The biggest advantage of SRM is the ability to probe chromatin organisation in its native cellular environment, allowing even live-cell chromatin dynamics studies. The high molecular specificity combined with the high resolution enable to detect the molecular composition of sub-diffraction sized compartments directly and without averaging.

The last decade significant advances with regard to super-resolution microscopes, (live-cell) labelling, and image analysis have been achieved, enabling to study chromatin at resolutions which allow to unravel its mechanistic functioning. For coordinate-targeted (e.g. STED) methods, these advances include dose-limiting acquisition strategies, non-toxic live-cell labelling methods, parallelisation, beam-shaping for 3D imaging, and labels for many-colour imaging. For stochastic methods (e.g. SMLM), these optimisations include the development of new labels, faster cameras, enhanced localisation-fitting algorithms, better drift correction, and extended multi-colour approaches.

One method not reviewed here, as it has not been applied to chromatin studies yet, is the newly developed MINimal fluorescence photon FLUXes (MINFLUX) (Balzarotti et al. 2017; Gwosch et al. 2020). MINFLUX is sometimes described as a hybrid technique that combines principles of both the coordinate-targeted and stochastic-localisation methods. Due to the unprecedented lateral and axial resolution of 1–3 nm both in fixed and living cells, and a temporal resolution of ~ 50 µs in single particle tracking, we expect this method to become of great value for chromatin research in the (near) future. After the initial development of MINFLUX, other approaches that all localise single emitting fluorophores have been developed. As these techniques are not (yet) commercially available, we refer to another review (Reymond et al. 2020) for a more in-depth method evaluation. As each technique has specific advantages and limitations, a careful consideration of the method of choice should be made based on the research question. Future technical developments in either the microscope, the labelling, or the analysis might require a reconsideration.

The emergence of strategies where SRM techniques are used in conjunction with other complementing methods is also promising for the chromatin field. Examples include the combination of expansion microscopy (ExM) with STED (fixed cells only) (Gao et al. 2018); SMLM combined with EM (srCLEM or srCryoCLEM) (Dahlberg and Moerner 2021; Derosier 2021; Jeong and Kim 2022); SRM with single-cell spatially resolved transcriptomics (Larsson et al. 2021); and utilising deep learning approaches for optimised SRM performance (Narayanasamy et al. 2022; Wang and Rivenson 2018). An example of a multi-modal imaging technique that has been specifically crafted for studying the chromatin structure is ChromSTEM (Li et al. 2022a) which has also been combined with STORM and Partial Wave Spectroscopy (PWS) in a platform called nanoscale chromatin imaging and analysis (nano-ChIA) (Li et al. 2021a).

With these recent technological advances, SRM is expected to become an even more important tool for investigating chromatin organisation under native cellular conditions at unprecedented spatial and temporal resolutions, providing a better understanding of how the chromatin structure enables its functioning and how important processes like transcription, replication, and repair are influenced by their localisation.

## Data Availability

Not applicable.
